# Differences in dietary patterns related to metabolic health by gut microbial enterotypes of Korean adults

**DOI:** 10.3389/fnut.2022.1045397

**Published:** 2023-01-06

**Authors:** Hwan-Hee Jang, Hwayoung Noh, Gichang Kim, Su-Yeon Cho, Hyeon-Jeong Kim, Jeong-Sook Choe, Jeongseon Kim, Augustin Scalbert, Marc J. Gunter, Oran Kwon, Hyesook Kim

**Affiliations:** ^1^National Institute of Agricultural Sciences, Rural Development Administration (NAS-RDA), Wanju, Jeollabuk-do, Republic of Korea; ^2^Nutrition and Metabolism Branch, International Agency for Research on Cancer (IARC-WHO), Lyon, France; ^3^Department of Cancer Prevention and Environment, Léon Bérard Cancer Center, L’Institut National de la Santé et de la Recherche Médicale (INSERM) U1296, Lyon, France; ^4^Department of Cancer Biomedical Science, Graduate School of Cancer Science and Policy, National Cancer Center, Goyang, Republic of Korea; ^5^Graduate Program in System Health Science and Engineering, Department of Nutritional Science and Food Management, Ewha Womans University, Seoul, Republic of Korea

**Keywords:** metabolic syndrome, gut microbiota, dyslipidemia, hyperglycemia, obesity

## Abstract

Diet has a profound impact on the progression of metabolic syndrome (MetS) into various diseases. The gut microbiota could modulate the effect of diet on metabolic health. We examined whether dietary patterns related to MetS differed according to gut microbial enterotypes among 348 Korean adults aged 18–60 years recruited between 2018∼2021 in a cross-sectional study. The enterotype of each participant was identified based on 16S rRNA gut microbiota data. The main dietary pattern predicting MetS (MetS-DP) of each enterotype was derived using reduced-rank regression (RRR) models. In the RRR models, 27 food group intakes assessed by a semi-quantitative food frequency questionnaire and MetS prediction markers including triglyceride to high-density lipoprotein cholesterol (TG/HDL) ratio and homeostatic model assessment for insulin resistance (HOMA-IR) were used as predictor and response variables, respectively. The MetS-DP extracted in *Bacteroides* enterotype (B-type) was characterized by high consumption of refined white rice and low consumption of eggs, vegetables, and mushrooms. The MetS-DP derived among *Prevotella* enterotype (P-type) was characterized by a high intake of sugary food and low intakes of bread, fermented legumes, and fermented vegetables. The MetS-DP of B-type was positively associated with metabolic unhealthy status (OR_*T*3 *vs*. *T*1_ = 3.5; 95% CI = 1.5–8.2), comparing the highest tertile to the lowest tertile. Although it was not significantly associated with overall metabolic unhealthy status, the MetS-DP of P-type was positively associated with hyperglycemia risk (OR_*T*3 *vs*. *T*1_ = 6.2; 95% CI = 1.6–24.3). These results suggest that MetS-DP may differ according to the gut microbial enterotype of each individual. If such associations are found to be causal, personalized nutrition guidelines based on the enterotypes could be recommended to prevent MetS.

## 1. Introduction

Metabolic syndrome (MetS) is characterized by a set of abnormal conditions such as abdominal obesity, hyperglycemia, dyslipidemia, and hypertension (1). MetS with insulin resistance and chronic low-grade inflammation leads to increased risks of diabetes and cardiovascular disease (CVD), which are major causes of mortality worldwide (2).

Modulation of dietary habits may be a major strategy for preventing MetS. Dietary patterns provide a comprehensive depiction of eating habits that can provide more information than an analysis of individual foods (3). Recent studies have provided scientific evidence for associations of dietary patterns with the prevention and pathogenesis of MetS (4, 5). Evidence from a meta-analysis of observational studies showed an inverse association between a “Healthy/Prudent” dietary pattern and risk of MetS, and a positive association between an “Unhealthy/Western” dietary pattern and the risk of MetS and its components (6). In the meta-analysis, “Healthy/Prudent” dietary patterns were characterized by higher intakes of fruit and vegetables, fish, and whole grains, whereas the “Unhealthy/Western” pattern was characterized by higher intakes of red meat, processed meat, refined grains, sweets, French fries, desserts, eggs, and high-fat dairy products (6).

In addition to the typical concept of “dietary patterns,” it is now necessary to consider an organism’s ability to process dietary components. The gut microbiota may have beneficial or detrimental effects on human health through the production of metabolites using dietary components as substrates (7). Diet is a key factor in determining the composition of the gut microbiome, and dietary patterns are associated with distinct combinations of gut bacteria (also called enterotypes) (8). The concept of enterotypes, proposed by Arumugam et al. (9), can be distinguished according to the dominant bacteria (*Bacteroides, Prevotella*, and *Ruminococcus*) in the fecal microbiome. Our previous study in Korean adults suggested that plant-and fermented food-based diets were positively associated with the *Ruminococcus* enterotype (10). Another study on the relationship between enterotypes and dietary patterns in Korean adults reported that a Korean-style balanced diet is potentially associated with *Ruminococcaceae* enterotypes at the family level and a reduced risk of MetS (11). Although a number of studies have investigated the relationships between dietary patterns and MetS risk, to the best of our knowledge, no study has investigated differences in dietary patterns associated with MetS according to enterotypes.

In the present study, therefore, we identified the main dietary pattern predicting MetS (MetS-DP) for each enterotype and investigated whether these enterotype-specific MetS-DP were associated with risk of metabolic unhealthy status including individual components of MetS. Our study potentially provides new insights into dietary patterns in relation to MetS depending on the diverse composition of the gut microbiota, which lays the groundwork for understanding and further exploring the concept of personalized nutrition to prevent MetS.

## 2. Materials and methods

### 2.1. Ethics statement

All participants provided written informed consent, and the study protocol was approved by the Public Institutional Review Boards of the Ministry of Health and Welfare, Korea (approval no. P01-202011-11-003) and by the IARC ethics committee (approval no. IEC 19-03-A1), and was registered at the Clinical Research Information Service (CRIS) of the Centers for Disease Control and Prevention of Korea (registration no. KCT0005676).

### 2.2. Study participants

This study pooled two cross-sectional studies developed in 2018 and 2021 as collaborative studies between the National Institute of Agricultural Sciences and the International Agency for Research on Cancer (NAS-IARC). Participants aged 18–60 years were recruited by the NAS from communities in Jeollabuk-do, Korea, for the first study (*n* = 179) from March to October 2018, and the second study (*n* = 172) between January and October 2021. Although 222 subjects were recruited in the first study (10), only 179 subjects, who agreed and provided additional written informed consent to use their biospecimen for other purposes beyond the previous study, were included in this study. For this study, we established a new cross-sectional study (Study 2) based on a previous cross-sectional study (Study 1) designed for other purposes where the gut microbiome data were available. The sample size of Study 1 was estimated based on its primary purpose and subjects were recruited by simple random sampling. Sample size of the current study was estimated based on Study 1. To investigate differences in metabolic status, we recruited more overweight and obese subjects in the new recruitment (Study 2) since the participants of Study 1 were relatively healthy subjects. The sample size of the new recruitment in Study 2 was estimated based on weight status as one of the metabolic status indicators by stratified random sampling. In the first study (10), volunteers were excluded if they were underweight or obese [body mass index (BMI) <18.5 kg/m^2^ or ≥30 kg/m^2^], had taken medications including antibiotics within the past 2 weeks, received hormone replacement therapy or used oral contraceptives within the past 2 weeks, were pregnant or breastfed within the past 6 months, or reported any disease such as metabolic disease, inflammatory bowel disease or cancer. In the second study, participants were recruited with the same exclusion criteria as in the first study, except for the inclusion of those who were obese (BMI ≥ 30 kg/m^2^) or who had any abnormal metabolic conditions such as hyperglycemia and dyslipidemia and were taking related medication. The study participants were initially invited to an information meeting a few days prior to the start of the study, where anthropometric data, including height and weight, were measured by trained research assistants, and exclusion criteria were ascertained. Of 351 eligible participants, the fecal sample of one participant failed to quality control and plasma biomarkers of two participants failed to be measured, leading to a sample size of 348 Korean adults (52% males) for this study.

### 2.3. Fecal sample collection, 16S rRNA sequencing, and gut microbial enterotype identification

We provided stool nucleic acid collection tubes (Norgen Biotek Co., Thorold, ON, Canada) to each participant. Stool samples were produced at home on the study day or the day before, stored at 4°C until brought to the study center, and then frozen at −18°C until further processing. DNA extraction from each fecal sample was performed using a PowerSoil^®^ DNA Isolation Kit (Cat. No. 12,888, MO BIO). The 16S rRNA amplicons covering variable regions V3-V4 were sequenced using the MiSeq platform (Illumina, San Diego, CA, USA). The resulting 16S rRNA gene sequences were analyzed using Quantitative Insights into Microbial Ecology (QIIME2) v2.2021.4 (12). The Divisive Amplicon Denoising Algorithm (DADA) two pipeline was applied to perform the quality control steps for the raw sequences, and then the amplicon sequence variant (ASVs) table was obtained. Taxonomic classification was assigned based on the NCBI BLAST database from the phylum to genus levels.

Enterotypes of the gut microbiota in the study participants were explored using a modified method to determine enterotype discovery in a previous study (10, 13) with a combination of principal coordinate analysis (PCoA) based on between-sample (β-) diversity indices (weighted UniFrac), followed by k-means cluster analysis based on the PCoA scores of the first two principal coordinates (PCos). The optimal number of clusters was determined by visual inspection of clusters derived using the silhouette (14) method (Supplementary Figure 1).

### 2.4. Dietary intake data collection and dietary pattern identification

Dietary intake data were collected using a semi-quantitative self-reported food frequency questionnaire (FFQ) developed and validated for the Korean diet by the Korea National Institute of Health (15). To recall food items or beverages consumed in the previous year, participants were asked to complete the FFQ table with inquiries about information on the average consumption frequency and serving size for 106 food items. Participants were given instructions, asked to fill out the FFQ, and return it on the study day. During the visit, trained research assistants reviewed the questionnaires with participants for completeness.

To identify dietary patterns, 106 food items were categorized into 27 food groups based on similarities in their nutrient composition as shown in Supplementary Table 1. The intake of food groups and macronutrients was calculated as grams per day (g/day) based on the consumption frequency and average portion size according to a food composition database established for the FFQ (15). The energy-adjusted daily intake of each food group was estimated using the residual method. Data on alcohol intake in the previous year were collected using a lifestyle questionnaire and converted into g/day. To identify MetS-DPs within each enterotype, reduced-rank regression (RRR) models were used to derive linear combinations of 27 food groups (predictor), maximizing the explained variability of MetS prediction biomarkers (response variables). The log-transformed plasma triglyceride to high-density lipoprotein cholesterol (TG/HDL-C) ratio and homeostatic model assessment for insulin resistance (HOMA-IR) were selected as response variables to predict MetS using the RRR model (16–18). The TG/HDL ratio has been proposed as an index of insulin resistance that increases cardiovascular risk and is known to be more closely related to coronary heart disease risk than sex, blood pressure, waist-hip ratio, and non-HDL cholesterol (19). HOMA-IR has the ability to estimate cardio-metabolic risk and serves as a powerful tool for the assessment of insulin resistance (20, 21). Food groups with factor loadings ≥| 0.2| were considered to have dominant contributions to distinctive dietary patterns (22, 23). The primary RRR dietary pattern of each enterotype was explained by 17.7% (for enterotype 1) and 13.7% (for enterotype 2) variation, whereas the secondary RRR dietary pattern was explained the 2.4% (for enterotype 1), and 10.4% (for enterotype 2) of variation (data not shown for secondary dietary pattern). To present the data concisely, we used the primary dietary pattern only for subsequent analyses because it explained the largest amount of variation in the response to MetS. The dietary pattern score was calculated as the sum of z-standardized energy-adjusted daily intake (mean = 0, standard deviation = 1) multiplied by an individual weight (factor loading) of each food group. Each participant received a factor score for the identified dietary pattern. These scores were used to rank participants according to their adherence to the dietary pattern.

### 2.5. Definition and measurements for metabolic health status

Individuals were diagnosed with metabolic unhealthy status if they met at least two of four diagnostic criteria for metabolic syndrome, not including blood pressure. The following criteria for each component suggested by the Korean Society for the Study of Obesity to define MetS (24) was used: abdominal obesity (waist circumference ≥90 cm for men and ≥85 cm for women), high plasma TG concentrations (≥150 mg/dL), low concentrations of plasma HDL cholesterol (<40 mg/dL for men and <50 mg/dL for women), and high concentrations of fasting plasma glucose (≥100 mg/dL).

Fasting blood samples were collected using a vacutainer (BD, Franklin Lakes, NJ) containing ethylenediaminetetraacetic acid (EDTA), and then centrifuged at 1,800 × *g* for 10 min to separate plasma. Collected plasma samples were aliquoted and stored at −80°C until further use. The levels of fasting glucose and TG were determined using a colorimetric assay kit (Sigma-Aldrich, St. Louis, MO, USA and Cayman Chemicals, Ann Arbor, MI, USA). The levels of C-reactive protein (CRP) were determined using an immunometric assay kit (Cayman Chemicals, Ann Arbor, MI, USA). HDL-C concentration was determined in the supernatant (HDL fraction) after precipitation of LDL using the HDL and LDL/VLDL cholesterol assay kit (Cell Biolabs Inc., San Diego, CA). Insulin levels were determined using an immunoassay kit (R&D Systems, Minneapolis, MN, USA). The levels of all plasma biomarkers were measured using a microplate reader (Molecular Devices Inc., Sunnyvale, CA, USA).

### 2.6. Statistical analysis

Data were expressed as percentages (categorical) or means ± standard error (continuous). The chi-square test for categorical variables and general linear models followed by the Tukey–Kramer test for continuous variables were used to examine the differences across the tertiles of MetS-DP scores. P-trends were assessed by the Mantel-Haenszel chi-square test for categorical variables or by modeling median values of the tertiles in linear regression models for continuous variables.

Multivariate logistic regression was used to calculate odds ratios (OR) with 95% confidence intervals (CI) to examine associations of MetS-DPs with risk of metabolic unhealthy status, after adjusting for covariates, including age (<30 vs. ≥30), sex, alcohol drinking status (yes vs. no), dietary supplement intake within 3 months prior to the enrollment (yes vs. no), regular physical activity (yes vs. no), smoking status (current vs. ever vs. never), education (<university graduation vs. <graduate school vs. ≥graduate school), menopause status (yes vs. no or men), medication use for metabolic abnormal condition (yes vs. no), and study sequence (Study 1 vs. Study 2). All statistical analyses were performed using the SAS (version. 9.4, SAS Institute) and R statistical software (version 4.1.1).

## 3. Results

### 3.1. Enterotypes in study participants

**FIGURE 1:**
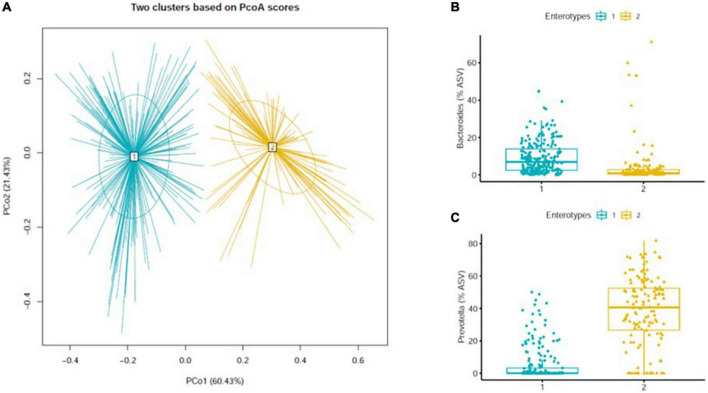
Enterotypes of gut microbiota based on weighted UniFrac distance matrix **(A)** and the two different enterotypes identified by one of the following dominant genera; *Bacteroides* **(B)**, and *Prevotella* **(C)**. Dominant bacteria genus of each enterotype were presented as relative abundance [proportion of the amplicon sequence variant (% ASV)].

Two enterotypes were identified in the study participants by PCoA and k-means clustering based on weighted UniFrac of the gut microbiota (Figure 1A). PCoA1 and PCoA2 had 60.4 and 21.4% of explained variance, respectively. At the genus level, the dominant bacteria of enterotype 1 and 2 were *Bacteroides* (Figure 1B) and *Prevotella* (Figure 1C), respectively. Of the total participants, enterotype 1 (B-type) and 2 (P-type) were 63 and 37%, respectively.

### 3.2. Dietary pattern predicting MetS according to enterotypes

The main MetS-DP within each enterotype derived by applying RRR is shown in Table 1. While high noodle intake and low leafy vegetable intake appeared as a common component for both MetS-DPs, overall, there were many differences between the two patterns. The B-type pattern was characterized by higher intake of refined white rice and lower intakes of eggs, starch vegetables, and mushrooms. The P-type pattern was characterized by higher intake of sugary foods and lower intakes of bread, fermented legumes, and fermented vegetables.

**TABLE 1:** Factor loadings of dietary patterns derived using reduced-rank regression and explained variation in food groups and response variables (TG/HDL-C ratio and HOMA-IR).

Food groups	B-type (*n* = 219)	P-type (*n* = 129)
Refined white rice	0.33	-0.14
Mixed grain rice	-0.12	0.16
Rice cake	-0.16	0.06
Cereal and snack	-0.06	-0.13
Bread	-0.02	-0.36
Noodle	0.30	0.28
Dumpling	0.13	0.27
Red meats	-0.01	0.13
Poultry	-0.22	-0.07
Fish	-0.14	-0.29
Other seafood	0.08	-0.07
Eggs	-0.39	-0.15
Non-fermented legumes	-0.17	-0.12
Fermented legumes	-0.04	-0.35
Fruit/fruit juice	-0.19	-0.22
Leaf vegetables	-0.36	-0.30
Starch vegetables	-0.31	0.03
Fruit vegetables	-0.28	-0.03
Fermented vegetables	0.01	-0.21
Other vegetables	0.06	-0.11
Mushroom	-0.24	0.09
Dairy products	-0.22	-0.16
Nuts and seeds	-0.05	-0.16
Coffee and tea	-0.04	-0.07
Coffee with sugar and cream	-0.18	0.24
Sugary beverage	-0.03	0.21
Confectionary and sweets	-0.03	-0.13
**Explained variation**		
Food groups (%)	7.10	3.86
Response variables (%)	17.72	13.68

### 3.3. Characteristics of the study participants according to tertiles of dietary pattern scores

Table 2 shows the differences in sex, age, BMI, education, drinking and smoking status, regular physical activity, dietary supplement intake, medication use for metabolic abnormal conditions, and menopause status of participants across tertiles of MetS-DP scores. In the B-type, participants with higher MetS-DP scores had a higher proportion of men (*P* for trend <0.001), lower age (*P* for trend = 0.008), a higher BMI (*P* for trend = 0.002), more alcohol drinkers (*P* for trend = 0.006) and smokers (*P* for trend = 0.007), and had lower dietary supplements (*P* for trend = 0.010). In the P-type, there was no significant trend in the participants’ characteristics across the tertiles of the MetS-DP score except for BMI and waist circumference. Participants with higher pattern scores had a higher BMI (*P* for trend = 0.009) and waist circumference (*P* for trend = 0.035). There was no statistically significant difference in all characteristics of the participants according to enterotypes.

**TABLE 2:** Characteristics of the study participants across tertiles of dietary pattern scores by enterotypes^1^.

B-type (*n* = 219)						P-type (*n* = 129)					*P*-value^3^
	**Total**	**T1**	**T2**	**T3**	***P*-trend^2^**	**Total**	**T1**	**T2**	**T3**	***P*-trend^2^**	
**Dietary pattern scores, range**	−3.0, 1.4	−3.0, −0.2	−0.2, 0.3	0.3, 1.4		−1.8, 1.6	−1.8, −0.2	−0.1, 0.2	0.2, 1.6		
**Sex**											
Men	109 (49.8)	18 (24.7)	37 (50.7)	54 (74.0)	<0.001	71 (55.0)	21 (48.8)	22 (51.2)	28 (65.1)	0.131	0.402
Woman	110 (50.2)	55 (75.3)	36 (49.3)	19 (26.0)		58 (45.0)	22 (51.2)	21 (48.8)	15 (34.9)		
**Age, y**											
<30	113 (51.6)	29 (39.7)	39 (53.4)	45 (61.6)	0.008	57 (44.2)	19 (44.2)	19 (44.2)	19 (44.2)	1.000	0.221
≥30	106 (48.4)	44 (60.3)	34 (46.6)	28 (38.4)		72 (55.8)	24 (55.8)	24 (55.8)	24 (55.8)		
**Waist circumference, cm**	80.3 ± 0.7	76.3 ± 1.1	79.9 ± 1.3	84.6 ± 1.3	<0.001	82.4 ± 0.9	80.8 ± 1.5	80.4 ± 1.7	85.9 ± 1.6	0.035	0.080
**BMI, kg/m^2^**	24.0 ± 0.3	23.1 ± 0.4	23.8 ± 0.5	25.2 ± 0.5	0.002	24.8 ± 0.4	24 ± 0.5	24.1 ± 0.6	26.3 ± 0.6	0.009	0.079
**Education**											
<University graduation	68 (31.1)	17 (23.3)	24 (32.9)	27 (37.0)	0.534	38 (29.5)	11 (25.6)	13 (30.2)	14 (32.6)	0.278	0.952
<Graduate school	80 (36.5)	37 (50.7)	20 (27.4)	23 (31.5)		48 (37.2)	15 (34.9)	16 (37.2)	17 (39.5)		
≥Graduate school	71 (32.4)	19 (26.0)	29 (39.7)	23 (31.5)		43 (33.3)	17 (39.5)	14 (32.6)	12 (27.9)		
**Drinking status**											
Yes	168 (76.7)	47 (64.4)	60 (82.2)	61 (83.6)	0.006	100 (77.5)	34 (79.1)	31 (72.1)	35 (81.4)	0.797	0.967
No	51 (23.3)	26 (35.6)	13 (17.8)	12 (16.4)		29 (22.5)	9 (20.9)	12 (27.9)	8 (18.6)		
**Smoking status**											
Current	28 (12.8)	5 (6.9)	7 (9.6)	16 (21.9)	0.007	18 (14.0)	6 (14.0)	4 (9.3)	8 (18.6)	0.139	0.415
Ever	27 (12.3)	8 (11.0)	10 (13.7)	9 (12.3)		22 (17.1)	5 (11.6)	6 (14.0)	11 (25.6)		
Never	164 (74.9)	60 (82.2)	56 (76.7)	48 (65.8)		89 (69.0)	32 (74.4)	33 (76.7)	24 (55.8)		
**Regular physical activity**											
Yes	117 (53.4)	45 (61.6)	37 (50.7)	35 (48.0)	0.098	64 (49.6)	22 (51.2)	19 (44.2)	23 (53.5)	0.830	0.564
No	102 (46.6)	28 (38.4)	36 (49.3)	38 (52.1)		65 (50.4)	21 (48.8)	24 (55.8)	20 (46.5)		
**Dietary supplement intake^4^**											
Yes	110 (50.7)	45 (62.5)	35 (48.6)	30 (41.1)	0.010	55 (42.6)	22 (51.2)	19 (44.2)	14 (32.6)	0.082	0.180
No	107 (49.3)	27 (37.5)	37 (51.4)	43 (58.9)		74 (57.4)	21 (48.8)	24 (55.8)	29 (67.4)		
**Drug use^4^**											
Yes	10 (4.6)	3 (4.2)	3 (4.2)	4 (5.5)	0.706	4 (3.1)	1 (2.3)	1 (2.3)	2 (4.7)	0.536	0.685
No	207 (95.4)	69 (95.8)	69 (95.8)	69 (94.5)		125 (96.9)	42 (97.7)	42 (97.7)	41 (95.4)		
**Menopause**											
Yes	16 (7.3)	8 (11.0)	5 (6.9)	3 (4.1)	0.113	7 (5.4)	3 (7.0)	0 (0.0)	4 (9.3)	0.635	0.647
No or men	203 (92.7)	65 (89.0)	68 (93.2)	70 (95.9)		122 (94.6)	40 (93.0)	43 (100.0)	39 (90.7)		

*T*, tertile. ^1^Values are mean ± SE or *n* (%). ^2^*P*-trend was assessed by modeling the median value of the tertiles in the linear regression analysis (continuous) or using the Mantel-Haenszel chi-squared test for linear trends (categorical),^3^differences between enterotypes were tested using a two-sample *t*-test (continuous) or using the chi-squared test (categorical). ^4^Missing values (*n* = 2) for non-responses.

### 3.4. Plasma biomarker according to tertiles of dietary pattern scores

As shown in Table 3, participants with higher pattern scores had higher HOMA-IR (*P* for trend <0.001 in both enterotypes), TG/HDL ratio (*P* for trend <0.001 in B-type and 0.004 in P-type), and higher levels of insulin (*P* for trend <0.001 in both enterotypes), CRP (*P* for trend = 0.002 in B-type and 0.082 in P-type), fasting glucose (*P* for trend <0.001 in B-type and 0.003 in P-type), and TG (*P* for trend <0.001 in B-type and 0.022 in P-type), and lower HDL cholesterol concentration (*P* for trend <0.001 for B-type and 0.023 for P-type). There were no significant differences between enterotypes in plasma metabolic diseases-related biomarkers.

**TABLE 3:** The concentration of plasma biomarkers across tertiles of dietary pattern scores by enterotypes^1^.

B-type (*n* = 219)						P-type (*n* = 129)					*P*-value^3^
	**Total**	**T1**	**T2**	**T3**	***P*-trend^2^**	**Total**	**T1**	**T2**	**T3**	***P*-trend^2^**	
**Fasting glucose, mg/dL**	108.1 ± 1.7	100.1 ± 2.7	109.7 ± 2.9	114.7 ± 3.1	<0.001	109.3 ± 2.3	101.9 ± 4.3	109.0 ± 2.9	117.1 ± 4.2	0.003	0.689
**Triglyceride, mg/dL**	133.2 ± 7.9	102.8 ± 8.3	123.1 ± 14.1	173.8 ± 16.3	<0.001	123.9 ± 6.3	111.0 ± 11.4	116.8 ± 8.8	143.7 ± 12.1	0.022	0.841
**HDL cholesterol, mg/dL**	37.6 ± 1.2	46.1 ± 2.2	36.1 ± 1.8	30.4 ± 2.0	<0.001	36.4 ± 1.5	39.6 ± 2.7	39.3 ± 2.8	30.1 ± 2.3	0.023	0.626
**Insulin, pmole/L**	36.2 ± 2.2	29.9 ± 2.3	34.7 ± 2.3	43.9 ± 5.8	<0.001	39.2 ± 3.1	28.3 ± 1.5	33.2 ± 2.3	56.0 ± 8.3	<0.001	0.308
**C-reactive protein, ug/mL**	0.8 ± 0.1	0.6 ± 0.1	0.8 ± 0.1	1.0 ± 0.1	0.002	0.9 ± 0.1	0.7 ± 0.2	0.6 ± 0.2	1.3 ± 0.3	0.082	0.662
**HOMA_IR**	1.7 ± 0.1	1.3 ± 0.1	1.6 ± 0.1	2.1 ± 0.3	<0.001	1.8 ± 0.2	1.2 ± 0.1	1.5 ± 0.1	2.8 ± 0.4	<0.001	0.318
**TG/HDL-C ratio**	5.4 ± 0.6	3.0 ± 0.4	5.2 ± 1.3	7.8 ± 0.9	<0.001	4.5 ± 0.3	3.9 ± 0.6	4.0 ± 0.5	5.7 ± 0.5	0.004	0.891

T, tertile. Log-transformed values were used for linear regression analysis and a two-sample t-test. ^1^Values are mean ± SE. ^2^*P*-trend was assessed by modeling the median value of the tertiles in the linear regression analysis, ^3^differences between enterotypes were tested using a two-sample *t*-test.

### 3.5. Nutrient intakes according to tertiles of dietary pattern scores

Table 4 shows the nutrient intake according to the tertiles of MetS-DP scores for each enterotype. There was no difference in the total energy intake in either enterotype or according to the tertiles of MetS-DP scores. The B-type MetS-DP score was positively associated with carbohydrate intake, while negatively associated with the intakes of protein fiber, zinc, β-carotene, riboflavin, vitamin B6, folate, vitamin C, vitamin E, and some flavonoids, such as anthocyanin isoflavones. The P-type MetS-DP score was marginally associated with intakes of alcohol (*P* for trend = 0.058) and total energy (*P* for trend = 0.071), while marginally inversely associated with intakes of flavonols (*P* for trend = 0.074) and anthocyanidins (*P* for trend = 0.051). Both MetS-DP scores were inversely associated with dietary intakes of total iron, vitamin A, vitamin B12, and vitamin D.

**TABLE 4:** Nutrient intakes across tertiles of dietary pattern scores by enterotypes^1^.

B-type (*n* = 219)						P-type (*n* = 129)					*P*-value^3^
	**Total**	**T1**	**T2**	**T3**	***P*-trend^2^**	**Total**	**T1**	**T2**	**T3**	***P*-trend^2^**	
**Carbohydrate%**											
<55	27 (12.3)	15 (20.6)	7 (9.6)	5 (6.9)	0.152	15 (11.6)	7 (16.3)	3 (7)	5 (11.6)	0.748	0.489
≥55 and <65	86 (39.3)	24 (32.9)	30 (41.1)	32 (43.8)		59 (45.7)	16 (37.2)	21 (48.8)	22 (51.2)		
≥65	106 (48.4)	34 (46.6)	36 (49.3)	36 (49.3)		55 (42.6)	20 (46.5)	19 (44.2)	16 (37.2)		
**Protein%**											
≥7 and <20	211 (96.4)	68 (93.2)	71 (97.3)	72 (98.6)	0.078	125 (96.9)	42 (97.7)	42 (97.7)	41 (95.4)	0.536	1.000
≥20	8 (3.7)	5 (6.9)	2 (2.7)	1 (1.4)		4 (3.1)	1 (2.3)	1 (2.3)	2 (4.7)		
**Total fat%**											
<15	28 (12.8)	7 (9.6)	10 (13.7)	11 (15.1)	0.057	22 (17.1)	6 (14)	9 (20.9)	7 (16.3)	0.835	0.211
≥15 and <30	177 (80.8)	58 (79.5)	59 (80.8)	60 (82.2)		94 (72.9)	32 (74.4)	31 (72.1)	31 (72.1)		
≥30	14 (6.4)	8 (11.0)	4 (5.5)	2 (2.7)		13 (10.1)	5 (11.6)	3 (7.0)	5 (11.6)		
Alcohol intake, g	9.0 ± 1.1	5.6 ± 1.2	12.5 ± 2.7	8.9 ± 1.5	0.168	9.7 ± 1.6	6 ± 1.0	9.7 ± 2.1	13.5 ± 4.2	0.058	0.709
Energy, Kcal	1,794 ± 47.9	1885.4 ± 97.4	1745.5 ± 78.2	1751.1 ± 71.4	0.231	1909.5 ± 89.8	1716.9 ± 84.6	1894.5 ± 127.8	2116.9 ± 220.0	0.071	0.258
Carbohydrate, g/1,000 kcal	166.7 ± 1.5	163.0 ± 3.2	167.1 ± 2.1	170 ± 2.2	0.049	165.6 ± 2.2	166.4 ± 3.2	167.8 ± 3.6	162.4 ± 4.4	0.477	0.645
Protein, g/1,000 kcal	39.4 ± 0.5	40.8 ± 1	38.8 ± 0.7	38.5 ± 0.6	0.043	39.0 ± 0.6	39.4 ± 0.9	38.4 ± 0.8	39.2 ± 1.2	0.857	0.583
Total fat, g/1,000 kcal	24.5 ± 0.4	25.5 ± 0.9	24.4 ± 0.6	23.6 ± 0.7	0.060	25.0 ± 0.7	24.7 ± 1	24.1 ± 1.2	26 ± 1.4	0.492	0.566
Dietary fiber, g/1,000 kcal	9.7 ± 0.2	11.1 ± 0.4	9.4 ± 0.3	8.7 ± 0.3	<0.001	9.2 ± 0.2	9.7 ± 0.4	9.0 ± 0.4	9.0 ± 0.3	0.136	0.093
Total iron, mg/1,000 kcal	7.2 ± 0.1	8.0 ± 0.2	7.1 ± 0.2	6.6 ± 0.2	<0.001	6.9 ± 0.1	7.3 ± 0.3	6.7 ± 0.2	6.7 ± 0.2	0.036	0.100
Zinc, mg/1,000 kcal	5.7 ± 0.1	5.9 ± 0.1	5.6 ± 0.1	5.5 ± 0.1	0.015	5.6 ± 0.1	5.6 ± 0.1	5.5 ± 0.1	5.5 ± 0.2	0.600	0.357
Vitamin A, RE/1,000 kcal	323.7 ± 10.5	398.2 ± 19.1	310.5 ± 17.2	262.3 ± 14.3	<0.001	307.5 ± 10.3	334 ± 19.4	305.9 ± 18.9	282.8 ± 14.5	0.042	0.274
β-carotene, ug/1,000 kcal	1542.2 ± 59.0	1887.4 ± 112.7	1486.4 ± 99	1252.9 ± 78.9	<0.001	1449.3 ± 59.3	1,575 ± 113.7	1429.4 ± 110.6	1343.4 ± 79.5	0.108	0.267
Thiamin, mg/1,000 kcal	0.6 ± 0	0.6 ± 0	0.6 ± 0	0.7 ± 0	0.755	0.7 ± 0	0.6 ± 0	0.7 ± 0	0.7 ± 0	0.017	0.665
Riboflavin, mg/1,000 kcal	0.6 ± 0	0.7 ± 0	0.6 ± 0	0.6 ± 0	<0.001	0.6 ± 0	0.6 ± 0	0.6 ± 0	0.6 ± 0	0.578	0.509
Niacin, mg/1,000 kcal	8.6 ± 0.1	8.9 ± 0.3	8.6 ± 0.2	8.2 ± 0.2	0.059	8.7 ± 0.2	8.5 ± 0.3	8.8 ± 0.4	8.8 ± 0.4	0.645	0.682
Vitamin B6, ug/1,000 kcal	0.8 ± 0	0.9 ± 0	0.8 ± 0	0.7 ± 0	<0.001	0.8 ± 0	0.8 ± 0	0.8 ± 0	0.8 ± 0	0.552	0.332
Folate, mg/1,000 kcal	252.0 ± 6.2	299.4 ± 11.0	239.3 ± 9.5	217.4 ± 9.2	<0.001	240.7 ± 6.3	255.4 ± 11.9	230.2 ± 10.0	236.5 ± 10.9	0.196	0.202
Vitamin B12, ug/1,000 kcal	4.0 ± 0.1	4.4 ± 0.2	4.1 ± 0.2	3.6 ± 0.2	0.008	3.9 ± 0.2	4.5 ± 0.3	3.6 ± 0.2	3.6 ± 0.3	0.013	0.550
Vitamin C, mg/1,000 kcal	53.7 ± 1.8	67.3 ± 3.5	50.7 ± 2.5	43.0 ± 2.8	<0.001	51.5 ± 2.1	53.5 ± 3.5	54.1 ± 4.2	47 ± 3.1	0.233	0.453
Vitamin D, mg/1,000 kcal	1.7 ± 0.1	2.1 ± 0.2	1.5 ± 0.1	1.4 ± 0.1	<0.001	1.6 ± 0.1	1.9 ± 0.1	1.6 ± 0.1	1.4 ± 0.1	0.012	0.678
Vitamin E, mg/1,000 kcal	5.0 ± 0.1	5.4 ± 0.1	4.8 ± 0.1	4.7 ± 0.1	<0.001	4.9 ± 0.1	4.9 ± 0.2	4.8 ± 0.2	4.9 ± 0.2	0.727	0.546
Flavan-3-ols, mg/1,000 kcal	9.8 ± 1.2	13.0 ± 1.7	9.4 ± 2.9	7.0 ± 1.4	0.044	9.2 ± 1.0	9.2 ± 1.8	8.4 ± 1.5	9.9 ± 2.0	0.797	0.708
Flavones, mg/1,000 kcal	0.2 ± 0	0.2 ± 0	0.2 ± 0	0.1 ± 0	0.178	0.1 ± 0	0.2 ± 0	0.1 ± 0	0.1 ± 0	0.671	0.440
Flavonols, mg/1,000 kcal	9.4 ± 0.4	12.3 ± 0.9	8.5 ± 0.5	7.4 ± 0.5	<0.001	8.5 ± 0.4	9.4 ± 0.6	8.2 ± 0.6	7.8 ± 0.6	0.074	0.087
Flavanones, mg/1,000 kcal	0.5 ± 0	0.6 ± 0.1	0.5 ± 0.1	0.4 ± 0.1	0.129	0.5 ± 0.1	0.4 ± 0.1	0.6 ± 0.1	0.5 ± 0.1	0.459	0.905
Anthocyanidins, mg/1,000 kcal	1.9 ± 0.1	2.8 ± 0.3	1.6 ± 0.2	1.2 ± 0.1	<0.001	1.8 ± 0.1	2.1 ± 0.2	1.8 ± 0.3	1.4 ± 0.2	0.051	0.507
Isoflavones, mg/1,000 kcal	12.5 ± 0.7	15.7 ± 1.5	12.1 ± 0.9	9.7 ± 0.7	<0.001	11 ± 0.6	12.1 ± 1.3	9.2 ± 0.7	11.6 ± 1.0	0.624	0.088

T, tertile. ^1^Values are mean ± SE or *n* (%). ^2^*P*-trend was assessed by modeling the median value of the tertiles in the linear regression analysis (continuous) or using the Mantel-Haenszel chi-squared test for linear trends (categorical). ^3^Differences between enterotypes were tested using a two-sample *t*-test.

### 3.6. Association between dietary pattern scores and risk of metabolic unhealthy status

Table 5 presents the associations between the MetS-DP scores and risk of metabolic unhealthy status and individual MetS components. In the crude model for the B-type, the MetS-DP score was positively associated with the prevalence of metabolic unhealthy status (referred to as two or more of the four components of MetS) and individual MetS components, excluding waist circumference. These associations remained in the model adjusted for age, sex, drinking status, dietary supplement intake, physical activity, smoking status, education, menopause, medication use for MetS, and study sequence. There was no consistent association between MetS-DP scores and MetS risk or individual components in the P-type. The MetS-DP score of the P-type was, however, significantly positively correlated with fasting blood glucose (*P* for trend = 0.008), even after adjustment for confounding factors.

**TABLE 5:** Association between dietary pattern scores and risk of metabolic unhealthy status by enterotypes^1^.

B-type (*n* = 219)					P-type (*n* = 129)			
	**T2 vs. T1**	**T3 vs. T1**	***P*-trend**	**Continuous (1-SD increase)**	**T2 vs. T1**	**T3 vs. T1**	***P*-trend**	**Continuous (1-SD increase)**
**Metabolic syndrome risk^2^**								
Crude	2.18 (1.12–4.22)	5.24 (2.52–10.91)	<0.001	3.21 (1.96–5.27)	0.91 (0.38–2.15)	2.47 (0.95–6.42)	0.071	2.06 (1.04–4.07)
Model1	1.42 (0.66–3.05)	3.45 (1.46–8.16)	0.005	2.47 (1.38–4.41)	0.83 (0.32–2.19)	2.48 (0.85–7.25)	0.121	2.12 (0.98–4.59)
Model2	1.42 (0.66–3.04)	3.45 (1.46–8.17)	0.005	2.46 (1.38–4.39)	0.81 (0.31–2.15)	2.4 (0.81–7.14)	0.142	2.09 (0.96–4.52)
**Waist circumference**								
Crude	1.10 (0.48–2.53)	2.12 (0.98–4.62)	0.051	1.87 (1.09–3.2)	0.61 (0.20–1.90)	2.02 (0.77–5.32)	0.129	1.98 (0.92–4.29)
Model1	0.68 (0.24–1.95)	1.50 (0.54–4.15)	0.339	1.7 (0.84–3.47)	0.48 (0.13–1.81)	2.93 (0.94–9.09)	0.071	2.54 (1.06–6.08)
Model2	0.68 (0.24–1.95)	1.50 (0.54–4.15)	0.342	1.7 (0.84–3.47)	0.43 (0.11–1.76)	2.90 (0.89–9.44)	0.082	2.58 (1.05–6.34)
**Fasting glucose**								
Crude	1.64 (0.85–3.17)	2.66 (1.35–5.24)	0.005	2.08 (1.34–3.21)	2.65 (1.10–6.43)	3.35 (1.35–8.31)	0.008	2.71 (1.34–5.51)
Model1	1.25 (0.54–2.93)	2.57 (1.05–6.27)	0.037	2.2 (1.20–4.04)	5.06 (1.31–19.58)	6.35 (1.63–24.67)	0.007	3.88 (1.43–10.52)
Model2	1.25 (0.54–2.93)	2.56 (1.05–6.27)	0.037	2.2 (1.20–4.04)	4.83 (1.24–18.85)	6.19 (1.58–24.25)	0.008	3.97 (1.44–10.97)
**Triglyceride**								
Crude	1.50 (0.62–3.63)	4.65 (2.06–10.48)	<0.001	3.96 (2.12–7.4)	1.00 (0.38–2.64)	1.9 (0.76–4.76)	0.161	1.92 (0.94–3.91)
Model1	1.07 (0.37–3.08)	3.50 (1.27–9.66)	0.007	4.26 (1.88–9.67)	1.15 (0.36–3.71)	3.35 (1.04–10.86)	0.044	2.95 (1.25–6.97)
Model2	1.06 (0.37–3.06)	3.50 (1.26–9.69)	0.007	4.26 (1.87–9.67)	1.13 (0.35–3.66)	3.26 (1.00–10.63)	0.050	2.9 (1.23–6.86)
**HDL-cholesterol**								
Crude	2.92 (1.46–5.87)	5.79 (2.63–12.74)	<0.001	3.14 (1.91–5.16)	1.11 (0.45–2.71)	2.02 (0.77–5.32)	0.158	1.73 (0.87–3.44)
Model1	2.54 (1.08–5.93)	5.71 (2.13–15.31)	<0.001	4.26 (1.88–9.67)	1.07 (0.39–2.93)	1.89 (0.64–5.56)	0.264	1.66 (0.76–3.63)
Model2	2.55 (1.09–5.96)	5.75 (2.14–15.45)	<0.001	2.77 (1.46–5.26)	1.07 (0.39–2.92)	1.88 (0.64–5.54)	0.269	1.65 (0.76–3.62)

T, tertile. ^1^Crude: not adjusted; Model 1: Adjusted for age, sex, drinking status, and dietary supplement intake (missing data = 2), regular physical activity, smoking status, education, menopause status, and study sequence. Model 2: Model 1 + additional adjustment for medication use for metabolic abnormal condition. ^2^Case subjects (*n* = 219) with two or more of the four metabolic syndrome components (waist circumference ≥90 cm for men and ≥85 cm for women, fasting glucose ≥100 mg/dL, triglycerides (≥150 mg/dL), and HDL cholesterol <40 mg/dL for men and <50 mg/dL for women), excluding blood pressure. Control subjects (*n* = 129) with 0 or 1 metabolic syndrome component.

## 4. Discussion

The purpose of the present study was to compare the dietary patterns derived by RRR method according to two enterotypes, and to investigate their association with risk of metabolic syndrome in Korean adults. We found that the MetS-DP differed by gut microbial enterotypes of individuals, even though other factors such as sex, age, BMI, other clinical markers, and nutrient intake did not differ across enterotypes.

Participants of the NAS-IARC cross-sectional study formed two distinct clusters, termed B-type (*Bacteroides*, 63%) and P-type (*Prevotella*, 37%), based on their dominant genera. Although the factors influencing enterotype clustering have not been clearly defined, a previous report stated that long-term diet, regardless of nationality, sex, age, and BMI, mainly affected the classification of *Bacteroides* and *Prevotella* enterotypes (25). In a recent large-scale microbiome study of 890 healthy Koreans, the participants were classified into two enterotypes: *Bacteroides* (60%) and *Prevotella* (40%) (26). Another study conducted among a Korean monozygotic twin population (*n* = 20) also identified two enterotypes dominant in *Bacteroides* or *Prevotella*, respectively, as shown in our study (13). A previous study, conducted in western populations, found a strong association between gut microbial enterotypes and long-term diet, suggesting that B-type was associated with animal protein and saturated fat intake, while P-type was associated with carbohydrate and simple sugar intake (25). However, in our study, there was no difference in dietary intake between the enterotypes except for some food groups (bread, non-fermented legumes, and sugary beverages) (Supplementary Table 2). The B-type participants had higher intakes of bread and non-fermented legumes and lower intakes of sugary beverages than the P-type participants. This result suggested that food components related to each enterotype could vary in different populations, so further studies with various populations with larger sample sizes are needed to confirm.

Although there was no significant difference in dietary intake between the two enterotypes, we investigated the difference in DPs predicting MetS according to the enterotypes. To identify a specific DP explaining metabolic health status in each enterotype, RRR analysis was used. RRR is a multivariate dimension reduction technique determining linear combinations of a set of predictor variables maximizing the explained variance in response variables (27). Unlike other DP analyses such as principal component analysis (PCA), RRR enables the identification of the DPs with optimal combinations of food groups (predictor variables) that could maximize the explained variance of the metabolic health parameters of interest (response variables) (28, 29). In the RRR models, TG/HDL ratio and HOMA-IR were included as response variables, as they have been demonstrated as powerful indicators of MetS (19–21). The explained variances of these response variables by the MetS-DPs identified in this study were 14–18%, which were relatively higher compared to other studies (3–12%) using biomarkers as response variables in RRR models (28, 30–32).

The MetS-DP in the B-type was characterized by high consumption of refined white rice and noodles, and low consumption of eggs and vegetables, while the MetS-DP of the P-type was characterized by high intakes of noodles, dumplings, and sugary beverages including coffee with sugar and low intakes of bread, fermented legumes, and leafy vegetables. In both enterotypes, the higher the MetS-DP score, the lower the intake of antioxidant nutrients, such as vitamin A, and vitamin B12. In addition, leafy vegetables (B-type = −0.36, P-type = −0.30) and fruits (B-type = −0.19, P-type = −0.22) of MetS-DPs showed negative loadings regardless of the enterotype. Previous studies have shown that intake of fruits and vegetables rich in antioxidants, folic acid, and flavonoids is associated with lower concentrations of systemic oxidative stress and inflammation (33–35) and lower MetS risk (36, 37). However, intakes of flavonols, anthocyanidins and isoflavones were inversely correlated with MetS-DP scores only in type B (Table 4). Many phytochemicals including isoflavones are selectively metabolized by gut microbiota (38). The gut microbial enterotype may modulate the relationship between diet and downstream metabolism. The dietary components associated with enterotype-dependent metabolites included artificial sweeteners, animal protein, and alcohol as well as plant-derived nutrients (fiber, carotenoids, and isoflavones) (39). Our results suggest that although there was no difference in nutrient intake, including macronutrients and dietary fiber, the metabolic activity of bioavailable components may vary depending on the properties of the gut bacteria composed in enterotypes.

We found that MetS-related variables (HDL-C, triglycerides, glucose, insulin, etc.) did not differ between the two enterotypes. However, our study showed that the MetS-DP of each enterotype had different associations with MetS risk. Only the B-type MetS-DP was associated with an increased risk of overall metabolic unhealthy status. Regarding the associations with individual MetS components, interestingly, both MetS-DP showed different tendencies. The B-type MetS-DP was associated with a risk of dyslipidemia, while the P-type MetS-DP was associated with a risk of abdominal obesity by waist circumference. Both MetS-DPs were associated with a risk of hyperglycemia. In the previous study, bile acids, a key regulator of lipid metabolism, showed different metabolic results depending on the enterotype (39). In a gut microbiome study among healthy adults where two enterotypes were defined by one dominant bacteria, *Ruminococcaceae*, plasma ursodeoxycholate (a bile acid) was positively correlated with metabolic and inflammatory indicators such as BMI and plasma CRP only in the *Ruminococcaceae*-dominant enterotype, not in another enterotype. Yet, the underlying mechanism between enterotypes and bile acid and lipid metabolism has not been elucidated, our study found a positive association of the MetS-DP with plasma CRP levels, and the risk of dyslipidemia, only in the B-type, not in the P-type. One animal study with individual species of Bacteroides showed that the human symbiont *Bacteroides thetaiotaomicron* exacerbates metabolic disorders by promoting lipid digestion and absorption (40).

Previous studies showed that the P-type had a higher capacity for degrading dietary fiber than the B-type (41) and a high *Prevotella*/*Bacteroides* ratio appeared more conducive to loss of body fat (42) and improvement of glucose metabolism (43) when individuals consumed diets high in fiber. Our finding that the P-type MetS-DP score was associated with risks of abdominal obesity and hyperglycemia supports previous evidence (42) that the populations with P-type were more responsive to the diet to body fat loss. In the P-type MetS-DP, the intakes of fermented legumes (factor loading = −0.35) and fermented vegetables (factor loading = −0.21) had negative correlations with MetS predictive indicators, such as TG/HDL ratio and HOMA-IR. The P-type was described as the enterotype that is predominantly found in non-Western populations that consume plant-based carbohydrates and fiber-rich ingredients as the main components of their diet (44). A recent intervention study also found that the traditional Korean balanced diet, which consisted of a low-glycemic diet containing kimchi and fermented soybeans, was more effective in women with P-type obesity (45). A fermented food diet also showed a noticeable impact on reducing inflammatory markers and increasing microbiota diversity in a prospective randomized study (46).

An Asian population-based cross-sectional study investigating the association between consumption of rice and noodles and MetS risk suggested that higher consumption of rice and noodles was associated with fasting blood glucose concentrations but not with systemic inflammation (47). This is consistent with the results of our study, which showed that the intake of noodles related to MetS-DPs for both enterotypes was positively related to the prevalence of hyperglycemia. However, in the P-type group, refined white rice consumption showed a negative factor loading (−0.14) with fermented foods, and this pattern showed a positive correlation with CRP. In a study of healthy Koreans without consideration of enterotype, greater consumption of rice was associated with a greater risk of MetS, whereas greater consumption of rice combined with kimchi, the main staple of the Korean diet, was associated with a lower risk of MetS (48). These results suggest that the effects of diet on MetS risk may differ based on interactions of food combinations with the gut microbiota.

To the best of our knowledge, this is the first study to identify the specific DP MetS-DP of each enterotype using RRR and then to investigate whether the MetS-DPs differ based on the gut enterotypes among Korean adults. However, several limitations of this study should be considered when interpreting the results. First, this study could not infer a causal relationship between MetS-DPs and enterotypes as it was a cross-sectional study. The cross-sectional study to which RRR was applied to derive dietary patterns could lead to an inverse causal relationship that the response variable may have influenced the reported food habits. Thus, it is necessary to confirm the causal relationship to the modulating factors through longitudinal studies and experimental approaches. Second, although a statistical model was used to adjust for the difference in participant characteristics between the two merged studies, there may have been selection bias. Third, antibiotic use affects gut microbial composition and diversity, and recent studies have shown that recovery of the gut microbiota after antibiotic exposure may take more than 2 weeks (49). However, there is a limit in this study to consider the effect of antibiotics due to the inability to collect data on the frequency of use of antibiotics. Finally, we could not investigate the association between MetS-DPs and blood pressure by enterotype because there were no blood pressure data available. Therefore, participants who met two or more of the four components of the diagnostic criteria for MetS were classified as the risk group.

## 5. Conclusion

In conclusion, our findings suggest that dietary factors associated with MetS risk may differ based on individuals’ gut microbiomes. In particular, among Korean adults, a refined rice-based diet in the B enterotype and a lower fermented food-based diet in the P enterotype were associated with an increased risk of MetS. Thus, further research, especially in prospective settings as well as experimental studies, is needed to confirm the results by limiting the possibility of reverse causation.

## Data availability statement

The raw data supporting the conclusions of this article will be made available by the authors, without undue reservation.

## Ethics statement

The studies involving human participants were reviewed and approved by the Public Institutional Review Boards of the Ministry of Health and Welfare, Korea and by the IARC Ethics Committee. The patients/participants provided their written informed consent to participate in this study.

## Author contributions

H-HJ and HN: methodology and investigation. H-HJ and HK: statistical analysis and writing – original draft. H-HJ, S-YC, and H-JK: conducting the field studies. JK: providing essential materials including dietary assessment tools. H-HJ, HN, J-SC, MG, and AS: conceptualization and funding acquisition. HN, GK, and OK: writing, review, and editing. HK: supervision. All authors contributed to the article and approved the submitted version.
