# Supplementary material for: Differences in dietary patterns related to metabolic health by gut microbial enterotypes of Korean adults
Source: Front Nutr. 2023 Jan 6;9:1045397. doi: 10.3389/fnut.2022.1045397 (PMC9853283; doi:10.3389/fnut.2022.1045397)
Supplement: Supplementary file 1 [file Image_1.pdf]

Supplemental Figure 1.

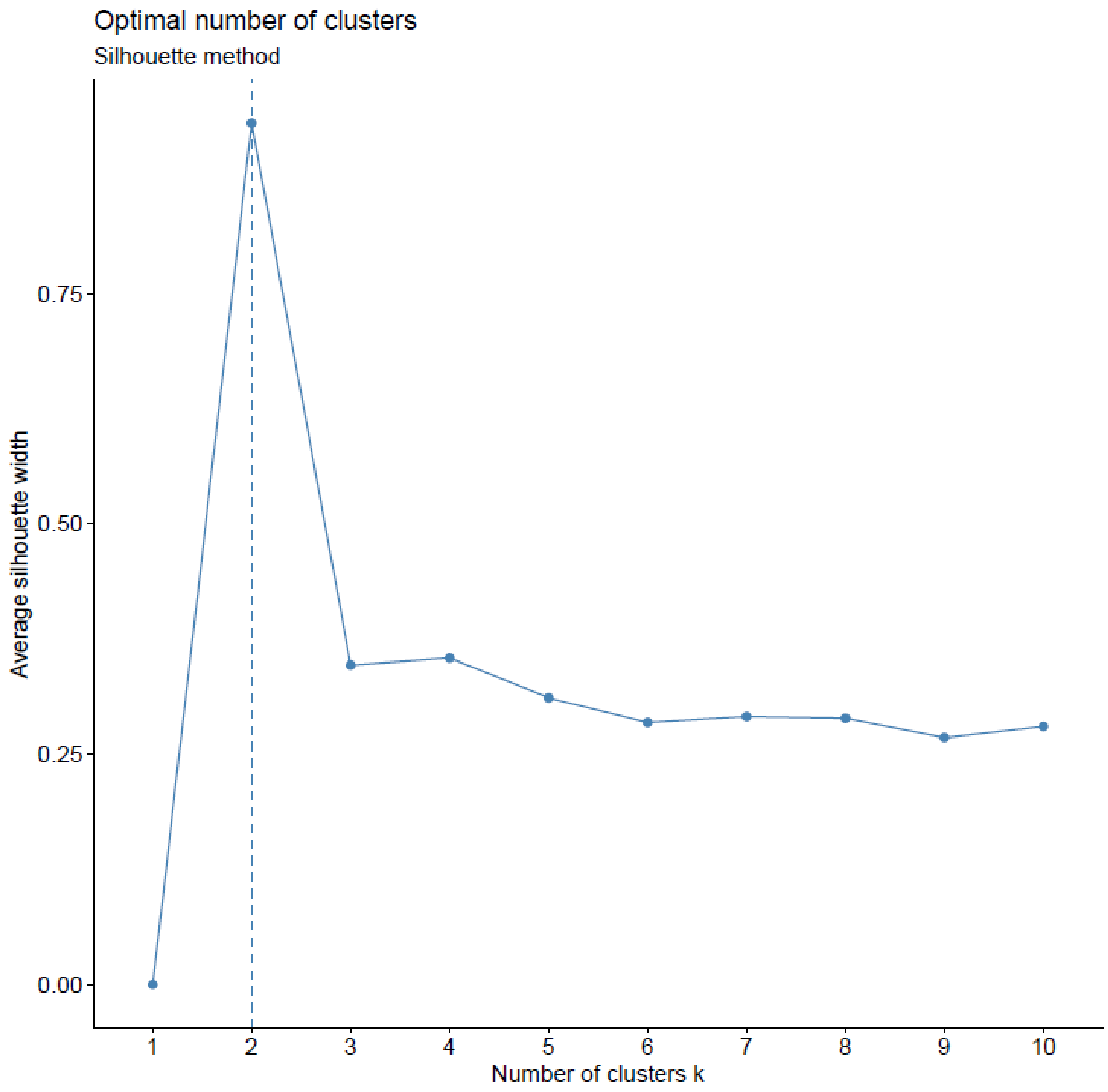

Figure S1. Plot to select optimal numbers of cluster with silhouette method on k-means clustering for identifying enterotypes on weighted UniFrac distance matrix.
