# Supplementary material for: Differences in dietary patterns related to metabolic health by gut microbial enterotypes of Korean adults
Source: Front Nutr. 2023 Jan 6;9:1045397. doi: 10.3389/fnut.2022.1045397 (PMC9853283; doi:10.3389/fnut.2022.1045397)
Supplement: Supplementary file 2 [file Table_1.docx]

**Table S1. Food grouping used in dietary pattern analysis**

| Food group(n=27) | Food Item (n=106) |
| --- | --- |
| Refined white rice | Cooked rice |
| Mixed grain rice | Cooked rice with soybeans, Cooked rice with cereals, Eat cooked rice/and cooked rice with soybeans almost same, Eat cooked rice/and cooked rice with cereals almost same |
| Rice cake | Rice cake (plain rod shape)/Rice cake with soup, Rice cake |
| Cereal and snack | Cookie/Cracker/Snack, Cereals/corn flakes |
| Bread | Loaf bread/Sandwich/Toast, Pizza/Hamburger, Bread with small red bean, Jam/Honey/Butter/Margarine (When you put on bread), Other bread |
| Noodle | Ramen, Wheat noodles with soup, Chajangmyon/Jambbong, Buckwheat vermicelli/Buckwheat noodle |
| Dumpling | Dumpling/Dumpling with soup |
| Red meats | Ham/Sausage, Pork(belly), Roasted pork, Braised pork, Edible viscera, Steak/Roasted beef, Dog meat, Beef soup, Beef soup with vegetables |
| Poultry | Fried chicken/Chicken stew |
| Fish | Sushi, Mackerel/Pacific saury/Spanish mackerel, Hair tail, Eel, Yellow croaker/Sea bream/Flat fish, Alaska pollack, Dried anchovy |
| Other seafood | Cuttlefish/Octopus, Salted-fermented fish, Clam/Whelk, Oyster, Crab, Shrimp, Fish paste/Crab flavored, Canned tuna, Dried laver, Kelp/Sea mustard |
| Eggs | Eggs/Quail eggs |
| Non-fermented legumes | Legumes (including beans/excluding cooked rice with bean), Tofu (including tofu in soups and stews), Soybean milk |
| Fermented legumes | Soup and Stew with soybean paste/Soybean paste |
| Fruit/Fruit juice | Peach/Plum, Banana, Persimmon/hard/Dried persimmon, Tangerine, Pear/Pear juice, Apple/Apple juice, Orange/Orange juice, Grape/Grape juice |
| Leaf vegetables | Korean cabbages/Korean cabbage soup, Spinach, Lettuce, Perilla leaf, Vegetables wrap/Vegetable salad, Other green vegetables, Pepper leaves/Chamnamul/Asterscaber, Crown daisy/Leek/Water dropwort |
| Starch vegetables | Starch jelly, Potato (including fried/steamed boiled potatoes), Sweet potatoes, Starch vermicelli |
| Fruit vegetables | Strawberry, Muskmelon/Melon, Watermelon, Tomato/Cherry tomato/Tomato juice, Cucumber, Green pepper, Immature pumpkin, Mature pumpkin/Pumpkin juice |
| Fermented vegetables | Kimchi/Korean cabbage, Kkakduki/Small radish kimchi, Kimchi/radish with water, Other kimchi (Green onion/Kodulbbagi/Mustard leaves), Korean style pickles |
| Other vegetables | Radish/Salted radish, Deoduck/Doraji (kind of white root), Bean sprouts, Bracken/Sweet potato stalk/Stem of taro, Carrot/Carrot juice, Onion |
| Mushroom | Oyster Mushroom, Other Mushrooms |
| Dairy products | Cheese, Yogurt, Milk |
| Nuts and seeds | Nuts, Parched cereal powder |
| Coffee and tea | Green tea, Coffee |
| Coffee with sugar and cream | Coffee with sugar, Coffee with cream |
| Sugary beverage | Carbonated drinks, Other types of tea or beverage |
| Confectionary and Sweets | Candy/Chocolate, cakes/chocopie, Ice cream |
