# Supplementary material for: Differences in dietary patterns related to metabolic health by gut microbial enterotypes of Korean adults
Source: Front Nutr. 2023 Jan 6;9:1045397. doi: 10.3389/fnut.2022.1045397 (PMC9853283; doi:10.3389/fnut.2022.1045397)
Supplement: Supplementary file 3 [file Table_2.docx]

Table S2. Food group intakes according to the enterotypes^1^

| Food group  (Kcal/total energy ratio) | B-type  (n=219) | P-type  (n=129) | P-value^2^ |
| --- | --- | --- | --- |
| Refined white rice | 16.5±1.4 | 14.6±1.7 | 0.4032 |
| Mixed grain rice | 21.2±1.4 | 22.1±2 | 0.6964 |
| Rice cake | 0.7±0.1 | 0.9±0.2 | 0.2363 |
| Cereal and snack | 3.3±0.3 | 3.3±0.4 | 0.9101 |
| Bread | 5.2±0.3 | 4.3±0.3 | 0.0411 |
| Noodle | 8±0.4 | 8.5±0.5 | 0.4474 |
| Dumpling | 0.7±0.1 | 0.7±0.1 | 0.6463 |
| Red meats | 12.8±0.4 | 14.1±0.8 | 0.1774 |
| Poultry | 1.5±0.2 | 1.3±0.1 | 0.1781 |
| Fish | 1.3±0.1 | 1.3±0.1 | 0.6297 |
| Other seafood | 1±0.1 | 1±0.1 | 0.7972 |
| Eggs | 1.9±0.1 | 1.8±0.2 | 0.6079 |
| Non-fermented legumes | 1.9±0.2 | 1.2±0.1 | 0.0022 |
| Fermented legumes | 1.4±0.1 | 1.2±0.1 | 0.3269 |
| Fruit/Fruit juice | 0.4±0 | 0.3±0 | 0.6197 |
| Leaf vegetables | 1.7±0.1 | 1.8±0.2 | 0.1746 |
| Starch vegetables | 4.4±0.3 | 4.2±0.4 | 0.7532 |
| Fruit vegetables | 1.3±0.1 | 1.3±0.1 | 0.0948 |
| Fermented vegetables | 0.9±0.1 | 0.9±0.1 | 0.8380 |
| Other vegetables | 1.2±0.1 | 1±0.1 | 0.7513 |
| Mushroom | 0.1±0 | 0.1±0 | 0.3137 |
| Dairy products | 4.3±0.3 | 4.1±0.3 | 0.5517 |
| Nuts and seeds | 0.8±0.1 | 0.8±0.1 | 0.7365 |
| Coffee and tea | 0.7±0.1 | 0.9±0.1 | 0.0552 |
| Coffee with sugar and cream | 0.4±0.1 | 0.6±0.1 | 0.2953 |
| Sugary beverage | 2.9±0.3 | 4±0.4 | 0.0253 |
| Confectionary and Sweets | 3.3±0.2 | 3.7±0.4 | 0.4421 |

^1^ Value are mean ± SE ^2^ Differences between enterotypes were tested using a two-sample t-test.
